# Supplementary figures and images for: A new perfusion culture method with a self-organized capillary network
Source: PLoS One. 2020 Oct 28;15(10):e0240552. doi: 10.1371/journal.pone.0240552 (PMC7592787; doi:10.1371/journal.pone.0240552)

Periphery

Center

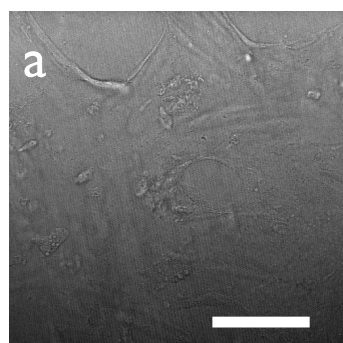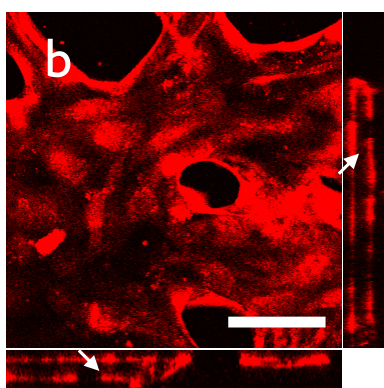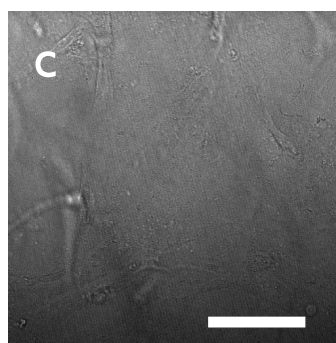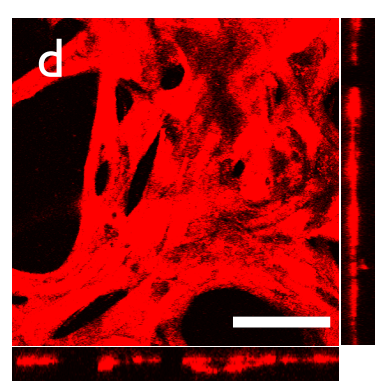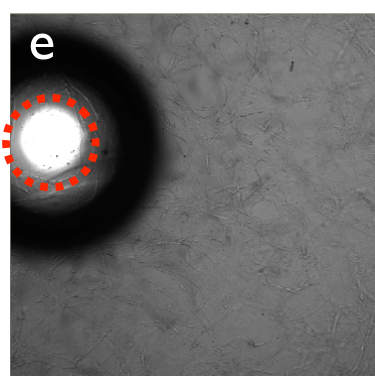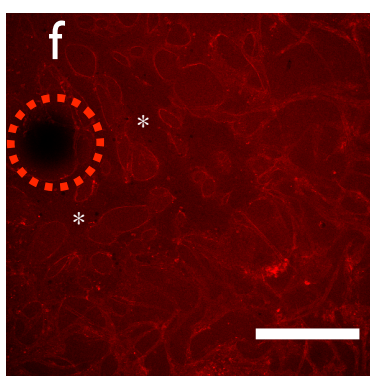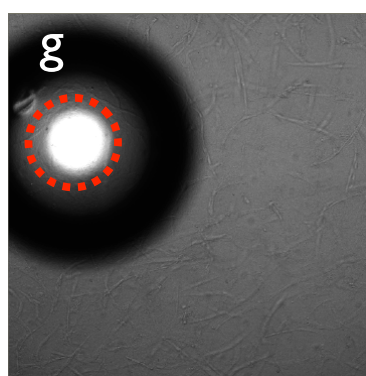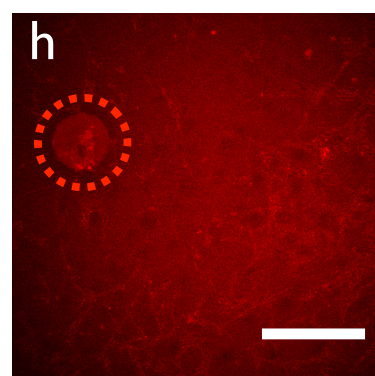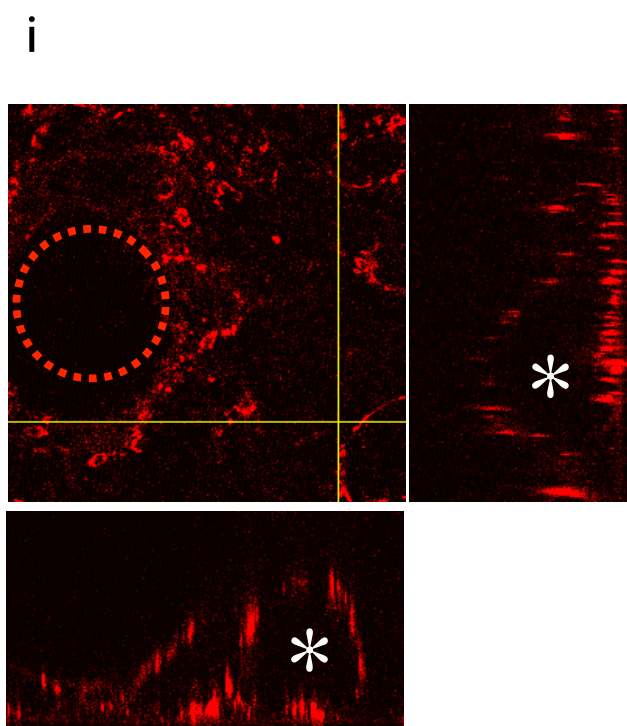

j

Normal dish      Dish with separator

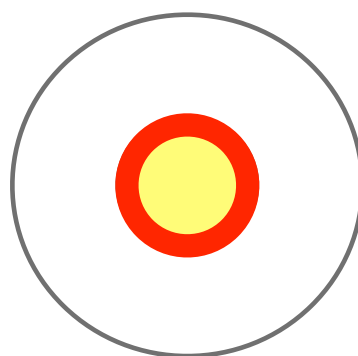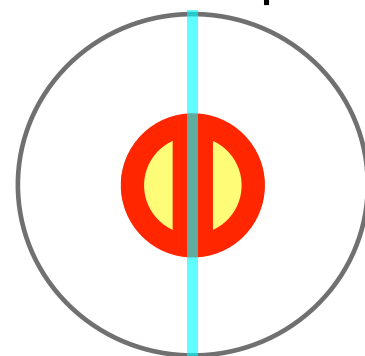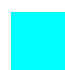

Glass separator

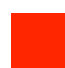

Lumen formation region

Supplement: S1 Fig — (a) Brightfield image of HUVECs cocultured with LFs in fibrin gel in the periphery of the dish. A lumen structure was observed. (b) Fluorescence image of HUVECs cocultured with LFs in fibrin gel in the periphery of the dish. HUVECs were stained with UEA1-FITC. A lumen structure was observed (white arrows). (c) Brightfield image of HUVECs cocultured with LFs in fibrin gel in the center of the dish. A lumen structure was not clear. (d) Fluorescence image of HUVECs cocultured with LFs in fibrin gel in the center of the dish. HUVECs were stained with UEA1-FITC. Lumen formation was not clear. (e) Brightfield image of HUVECs cocultured with LFs in fibrin gel near 1 mm glass beads (dashed red circle) embedded in the fibrin gel. We observed lumen formation around beads. (f) Confocal image of HUVECs cocultured with LFs in fibrin gel near 1 mm glass beads embedded in the fibrin gel. HUVECs were stained with UEA1. We observed lumen formation around beads. (g) Brightfield image of HUVECs cocultured with LFs in fibrin gel near 1 mm glass beads placed on the fibrin gel. We observed lumen formation around beads. (h) Confocal image of HUVECs cocultured with LFs in fibrin gel near 1 mm glass beads placed on the fibrin gel. HUVECs were stained with UEA1. We observed lumen formation around the beads (asterisks). (i) Three-dimensional structure of RFP-HUVECs around the glass beads. We observed the lumen around beads (asterisks). (j) Scheme of lumen formation region in normal glass-bottom dish and dish with glass separator. Scale bars: 100 μm (a–d, i); 1 mm (e-h). (PDF) [file pone.0240552.s001.pdf]

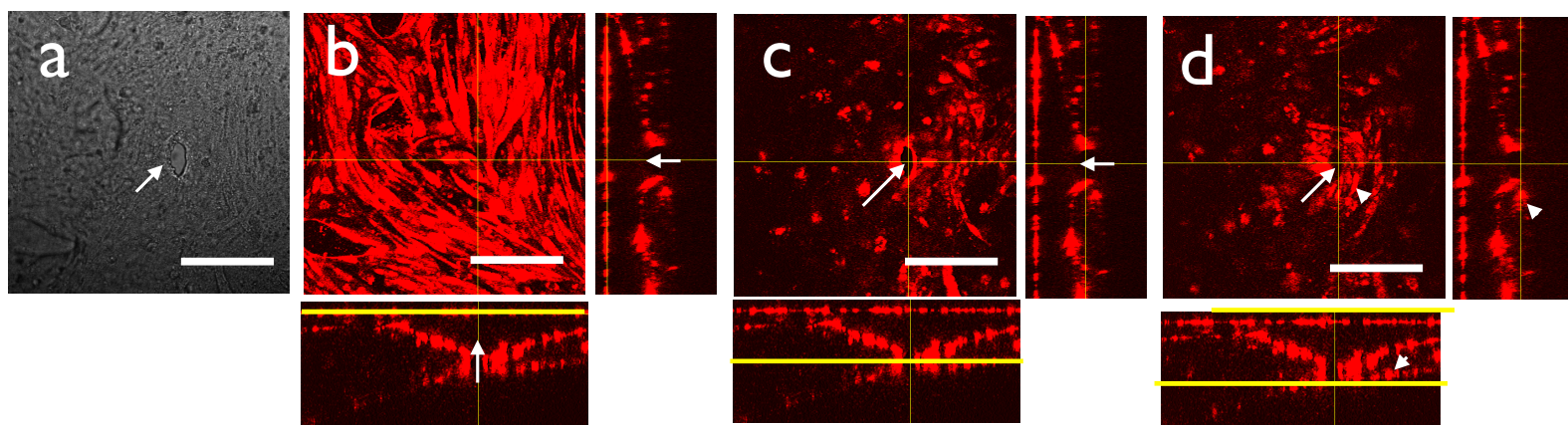

Supplement: S2 Fig — (a) Brightfield image of the inlet hole region. Arrow: small hole made by fine forceps. (b) Three-dimensional structure of the inlet hole observed by confocal microscopy, basal level. We observe RFP-HUVECs formed the floor of the lumen near the glass bottom. A hole at the ceiling was observed (arrow). (c) Three-dimensional structure of the inlet hole observed by confocal microscopy, middle level. The hole is bordered by RFP-HUVECs (arrow). (d) Three-dimensional structure of the inlet hole observed by confocal microscopy, upper level. The hole was continuous (arrow), and endothelial cells covered the surface of the fibrin gel (arrowheads). Scale bars: 200 μm. (PDF) [file pone.0240552.s002.pdf]

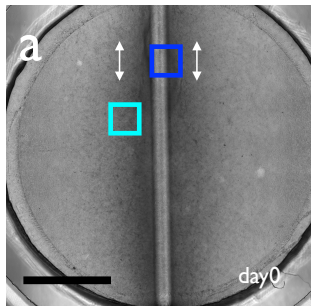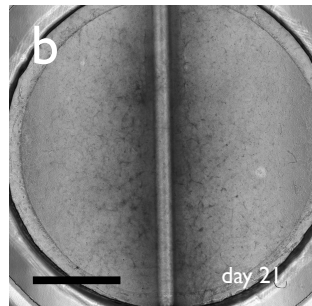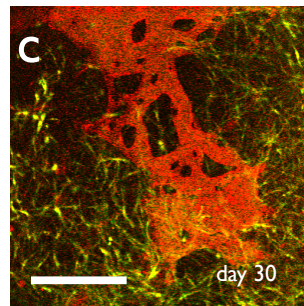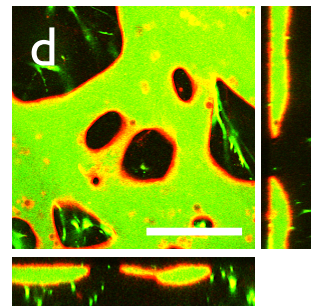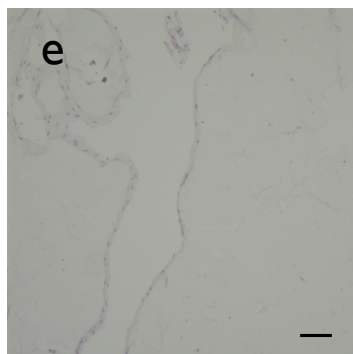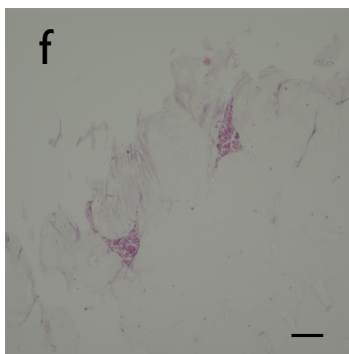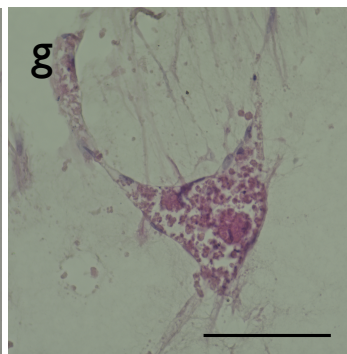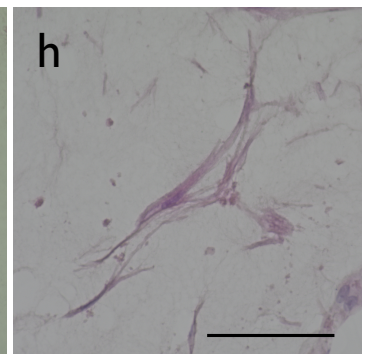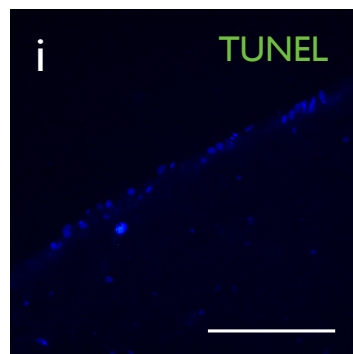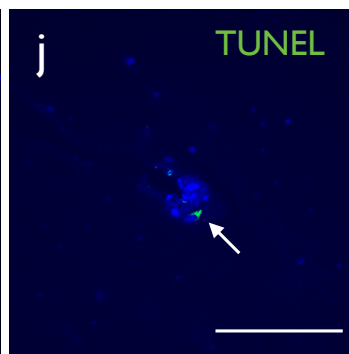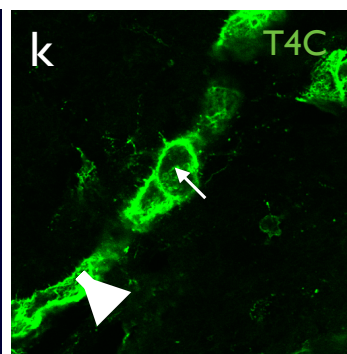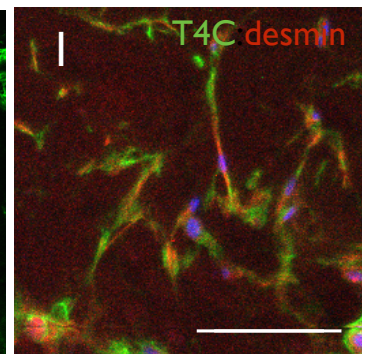

Supplement: S3 Fig — (a) Low magnification view at day 0. Locations of the inlet and outlet are shown by white arrows. (b) Low magnification view at day 21. (c) Confirmation of perfusion at day 30. GFP-pericytes were labeled green. Endothelial cells were stained with UEA-1 lectin (red). Characteristics of the vessel shape were similar to that without pericytes. (d) Three-dimensional view of the vasculature. We perfused culture medium with FITC-dextran (green) and did not observe leakage. (e) Histological observation of the flow region. (f) Histological observation of the low flow region. (g) High magnification view of the cyst structure in the non-flow region. (h) Histological structure of pericytes within a gel in the non-flow region. (i) TUNEL staining of the flow region. No dead cells were observed. (j) TUNEL staining of the non-flow region. A positive signal was observed within the cyst. (k) Type IV collagen (T4C) staining near the cyst. A large ECM sheath structure was observed (arrowhead) near the cyst (arrow). (l) Type IV collagen and desmin staining of the non-flow region. Desmin-positive pericytes were observed within the gel, which colocalized with type IV collagen. Scale bars: 3 mm (a, b); 1 mm (c); 250 μm (d);100 μm (e-l). (PDF) [file pone.0240552.s003.pdf]

a

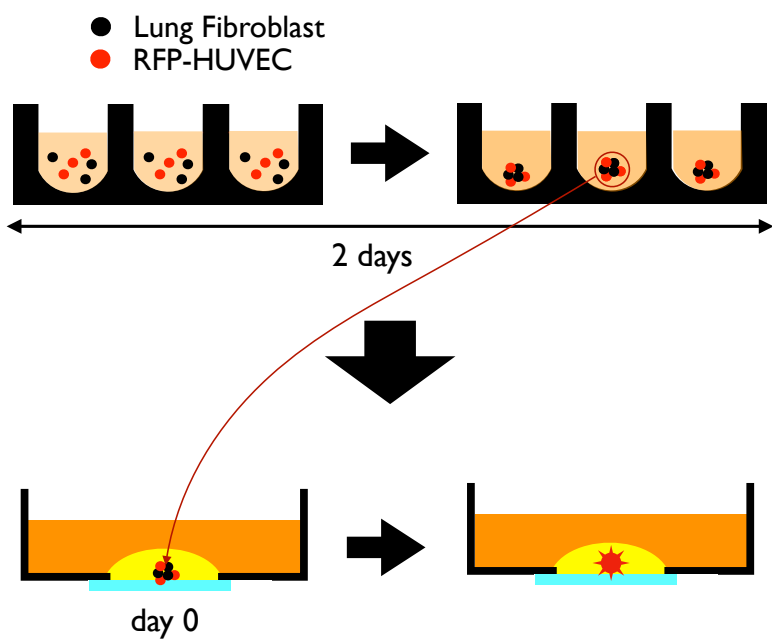

b

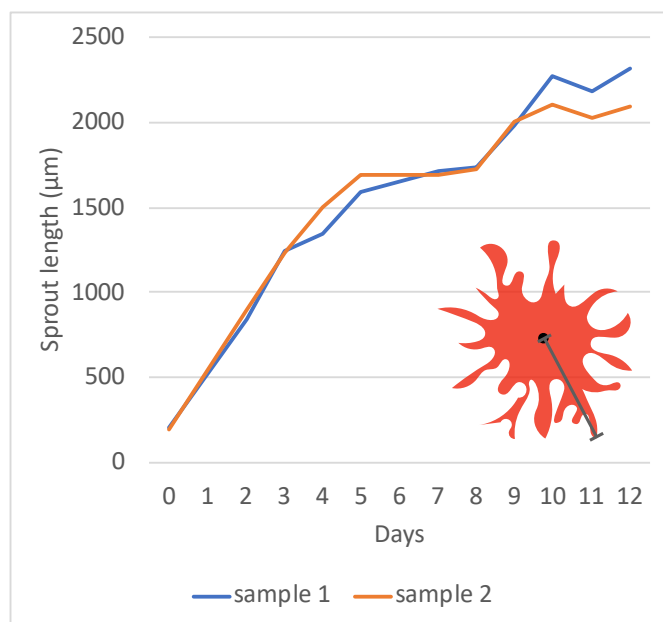

Supplement: S4 Fig — (a) Spheroids containing RFP-HUVECs were cultivated for 2 days in a culture plate and then transferred to fibrin gel. (b) The length of angiogenic sprouts from the centroid of the fluorescent signal was measured every day. The length of the sprout became saturated after 7 days, and the radius was around 1 mm, which enabled us to directly cut the tip of the sprout to generate an open end. (PDF) [file pone.0240552.s004.pdf]

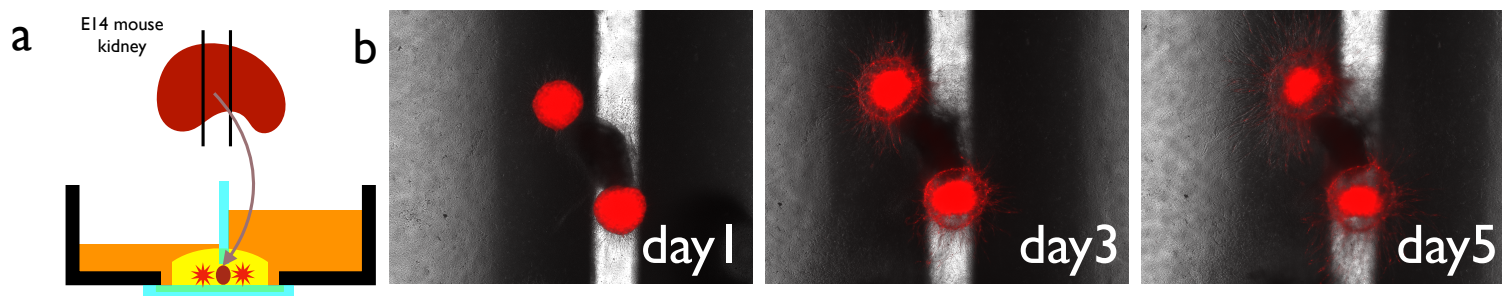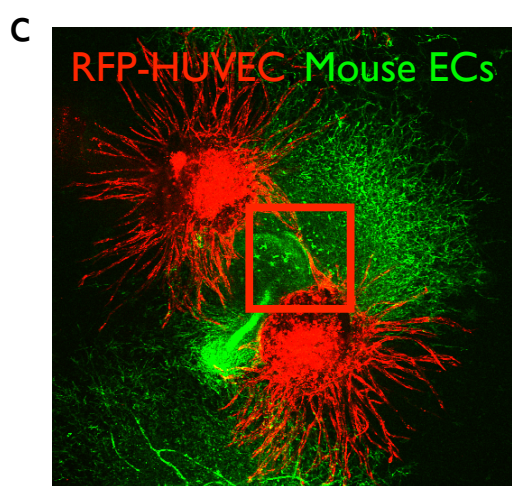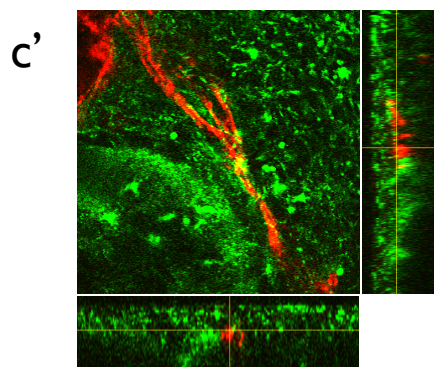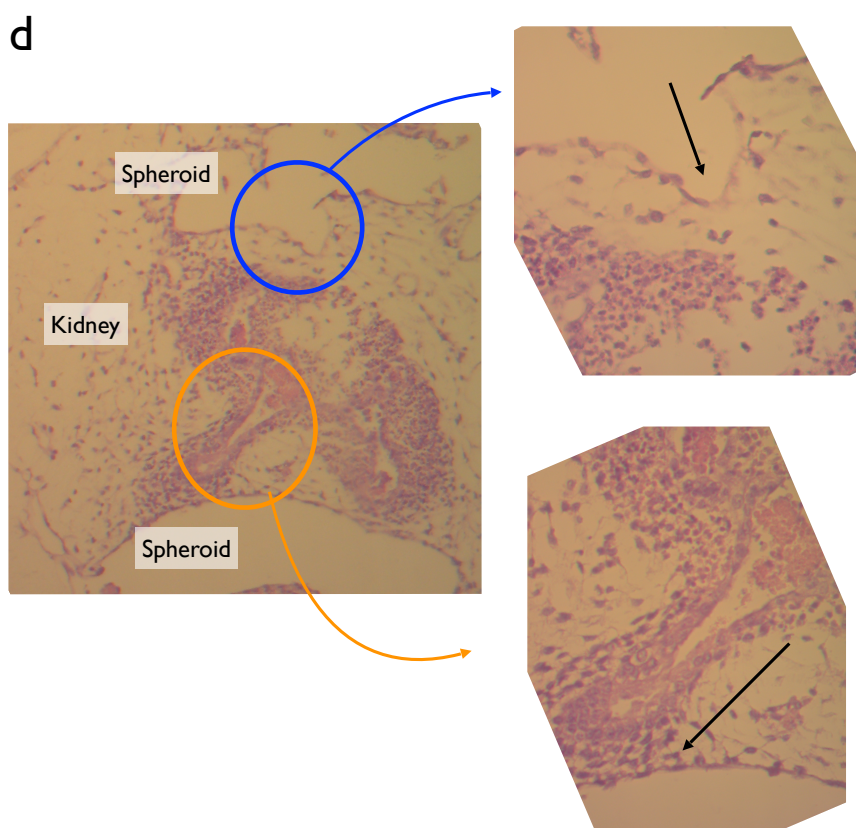

Supplement: S5 Fig — (a) E12 mouse embryonic kidney tissue was dissected, and the central one-third of the kidney was embedded in fibrin gel. The tissue was sandwiched by two RFP-HUVEC:hLF spheroids. (b) After 1 week, HUVEC-LF spheroids generated sprouts towards the embryonic kidney tissue. (c) Sprouts from RFP-HUVECs appeared to avoid the embryonic tissue. (c’) 3D observation of HUVECs and mouse endothelial cells revealed that, although mouse endothelial cells (green) tended to attach to RFP-HUVECs, they did not connect vasculature with lumens together. (d) Histological observation of the cultured tissue. Kidney explants formed collecting tubule-like structures, but the endothelial sprouts from the HUVEC spheroids did not form a connection with the kidney structure. (PDF) [file pone.0240552.s005.pdf]

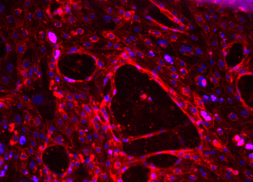

Supplement: S4 Movie — Time-lapse movie of the flow region in the long-term flow experiment (Fig 2A–2C). The stream-like movement of endothelial cells was observed in the opposite direction to flow. Frame rate: 3 min/frame, 180 min. (TIF) [file pone.0240552.s009.tif]
